# Identifying Clinical Predictors of Unfavorable Treatment Trajectories in Major Depressive Disorder: A National Multicentered Prospective Cohort Longitudinal, Naturalistic Study

**DOI:** 10.1155/da/9897990

**Published:** 2026-07-17

**Authors:** Xuequan Zhu, Kaiyan Gan, Xu Chen, Lei Feng, Yuan Feng, Ling Zhang, Gang Wang

**Affiliations:** ^1^ Beijing Key Laboratory of Intelligent Drug Research and Development for Mental Disorders, National Clinical Research Center for Mental Disorders, National Center for Mental Disorders, Beijing Anding Hospital, Capital Medical University, Beijing, China, ccmu.edu.cn

**Keywords:** antidepressant, longitudinal trajectories, major depressive disorder, side-effect burden, treatment outcomes

## Abstract

**Background:**

While antidepressant side effects are often expected to diminish with continued treatment, empirical evidence remains limited. This study investigates co‐trajectories of depressive severity with side‐effect burden (SEB) and associated factors in patients with major depressive disorder (MDD).

**Methods:**

We analyzed longitudinal data from 1377 MDD outpatients across three Chinese multicenter studies (2015–2022), with assessments at baseline, weeks 2, 4, 8, 12, and 24. Depression severity was measured using the Quick Inventory of Depressive Symptomatology‐Self Report (QIDS‐SR16). At the same time, SEB was quantified using the frequency, intensity, and burden of side effects rating (FIBSER). Dual‐trajectory modeling was used to identify distinct SEB and depression severity trajectories, with linear mixed‐effects models comparing depression changes across SEB groups.

**Results:**

Four SEB trajectories were identified: no SEB (41.5%), early‐onset SEB (29.1%), late‐onset SEB (14.7%), and persistent SEB (13.9%). Depressive severity followed three trajectories: mild‐responsive (66.6%), moderate‐progressive (18.7%), and chronic‐severe (14.7%). Persistent SEB was associated with higher baseline depression severity (OR = 1.13, 95% CI: 1.08–1.17), antidepressant combination (OR = 3.7, 95% CI: 1.52– 9.02), and poorer treatment outcomes. 7.3% exhibited concurrent chronic‐severe depressive severity and persistent SEB. Female (OR = 1.95, 95% CI: 1.11–3.42), younger age (OR = 5.02, 95% CI: 2.63–9.55), higher education (high school: OR = 2.5, 95% CI: 1.12–5.36; bachelor and above: OR = 2.68, 95% CI: 1.24–5.82), and combination antidepressant (OR = 8.27, 95% CI: 2.69–25.45) were significant risk factors for concurrent severe symptoms and persistent SEB.

**Conclusion:**

Persistent antidepressant side effects coevolve with unfavorable depression trajectories over 6 months. Clinicians should prioritize early monitoring and tailored interventions for high‐risk subgroups, particularly those with severe baseline symptoms or on combination therapy. These findings underscore the importance of continuous monitoring and personalized interventions to manage antidepressant side effects effectively.

## 1. Introduction

As the cornerstone pharmacological intervention for major depressive disorder (MDD), antidepressant medications demonstrate well‐established efficacy and acceptable tolerability in randomized controlled trials [1]. Current treatment guidelines advocate dose optimization through progressive titration to individual tolerance thresholds [2, 3], reflecting the well‐documented association between treatment persistence and tolerability rather than efficacy parameters [4, 5]. However, real‐world effectiveness is substantially limited by side profiles, with only 41.3% of patients completing an acute treatment phase without experiencing medication‐related side effects, and, on average, each patient reports 2.9 side effects [6]. The rapid onset of adverse effects creates a critical vulnerability period for nonadherence and early treatment termination [7, 8] and may substantially impact the long‐term prognosis [9].

While numerous studies have reported antidepressant side effects in patient demographics [10], clinical characteristics [11], and pharmacological profiles [12], there remains a notable lack of longitudinal data on their temporal evolution during sustained treatment [13, 14]. This knowledge gap is particularly concerning given findings from the Netherlands Study of Depression and Anxiety (NESDA), which contradict the common clinical expectation that side effects diminish with continued medication use, revealing that patient‐perceived side effects persist unchanged throughout long‐term antidepressant treatment [6].

Moreover, despite well‐established data on side‐effect prevalence, few studies have employed longitudinal designs to investigate the dynamic relationship between side effects and disease progression [13, 15]. The field has largely neglected the critical interaction between evolving side‐effect profiles and treatment outcomes. At the same time, clinicians often work under the impression that the side‐effect burden (SEB) diminishes as patients adjust to the medication; however, this premise warrants further empirical investigation [16, 17]. This oversight may lead to suboptimal treatment adjustments, reduced adherence, and prolonged illness duration. Importantly, prior evidence from the International Study to Predict Optimized Treatment for Depression (iSPOT‐D) study suggests that the functional burden of side effects, rather than their frequency or intensity alone, may be more closely associated with poorer antidepressant outcomes [7]. However, the longitudinal interplay between SEB and depressive severity during treatment is complex, and the specific predictors that identify patients at risk for poor outcomes are not well established.

To better understand this problem, we used pooled data from three large, multicenter prospective studies of outpatients with MDD. We hypothesized that the burden of side effects would coexist with unfavorable depressive trajectories over follow‐up. We aimed to first characterize the joint trajectories of depressive severity and SEB as they evolve together during the initial months of antidepressant treatment. Following that, we sought to identify key baseline demographic and clinical factors that predict which patients are likely to experience persistent side effects and poor treatment responses. By identifying these high‐risk groups, our study provides clinicians with an empirical basis for early patient stratification and for developing more personalized approaches to improve treatment outcomes.

## 2. Methods

### 2.1. Participants and Settings

The current study pooled data from three studies conducted between 2015 and 2022, all of which enrolled patients with MDD initiating antidepressant treatment for their current episode and implemented similar eligibility criteria and follow‐up protocols: (1) the study of measurement‐based care treatment of MDD conducted from April 2015 to December 2017 at 11 psychiatric hospitals or units in general hospitals in nine cities in China; (2) Appropriate technology study of MDD diagnosis and treatment based on objective indicators and measurement conducted from September 2016 to December 2020 at eight psychiatric hospitals or units in general hospitals in 8 cities in China [18]; (3) Prospective cohort study to examine the Effect of Initial Treatment choice for depressive episodes conducted from August 2020 to December 2022 at 17 psychiatric hospitals and 10 general units in general hospitals in 19 cities of China [19]. In general, inclusion criteria for the current study included (1) Outpatients who were aged 18 years and above; (2) patients with depressive episodes, recurrent depressive disorder based on the Diagnostic and Statistical Manual of Mental Disorders, 5th Edition (DSM‐5) or The International Statistical Classification of Diseases, 10th Revision (ICD‐10); (3) patients initiated treatment for the current depressive episode; and (4) patients who had moderate and severe symptoms. Patients with low adherence, as determined by the researchers, were excluded. Scheduled follow‐up assessments were at baseline, weeks 2, 4, 8, 12, and 24. In the current study, we included patients with at least a week 2 follow‐up assessment.

All patients provided written informed consent to participate in the study before any procedures were conducted. All procedures in each study comply with the ethical standards of the Helsinki Declaration and the International Conference on Harmonization Good Clinical Practice guidelines. The independent ethics committee of each study site approved the studies.

### 2.2. Measures

#### 2.2.1. Depressive Severity

In all three studies, participants completed the Quick Inventory of Depressive Symptomatology‐Self Report (QIDS‐SR16) [20, 21] as a measure of depressive severity. QIDS‐SR16 consists of 16 items, each rated on a 0–3 scale. The total score ranged from 0 to 27, with higher scores indicating more severe depressive symptoms. QIDS‐SR16 was administered at baseline and at each follow‐up time point.

#### 2.2.2. Global SEB

In our analysis, SEB was indexed by the third item of the frequency, intensity, and burden of side effects rating ( FIBSER ) scale [22]. The scale was developed to document side effects in patients treated with antidepressants, capturing three global dimensions (frequency, intensity, and burden) and scored on a 7‐point scale. The burden item defined SEB as “choose the response that best describes the degree to which antidepressant medication side effects that you have had over the last week have interfered with your day‐to‐day functions,” with those who had mild or severe impairment as having a global SEB (labeled group = 1) and those who reported no impairment or minimal impairment as not having a SEB (labeled group = 0). This dichotomization was used to ensure comparability across the pooled naturalistic cohorts and to model longitudinal SEB trajectories as a binary outcome using a logit specification. As one of the studies (the third) used in the current analysis exclusively assessed the third item of the FIBSER, we used only this item as the global SEB metric. The item of SEB was measured at baseline and at each point of the follow‐up period (see Supporting Information 1: Table S1 for the item description).

#### 2.2.3. Clinical Characteristics

The pharmacological treatment of the depressive episode in this study was recorded at baseline and at each follow‐up visit. It was further categorized as a selective serotonin reuptake inhibitor (SSRI), a selective serotonin –norepinephrine reuptake inhibitor (SNRI), other antidepressants, and antidepressant combinations. Other antidepressants are referred to non‐SSRI and non‐SNRI antidepressant monotherapy. In regression analyses, other antidepressant monotherapies were used as the reference category. For sample representativeness and statistical stability, the antidepressant combination was not further stratified.

### 2.3. Statistical Method

Demographic and clinical characteristics were summarized by descriptive statistics. For quantitative variables, one‐way analysis of variance ( ANOVA ) was performed, followed by post‐hoc comparisons using the Newman –Keuls test when the main effect indicated pairwise differences between groups. For categorical variables, we used the CMH test, and a post‐hoc Newman –Keuls test was used for pairwise comparisons.

We employed a dual‐trajectory model to analyze the longitudinal patterns of the SEB and depressive severity. This approach allows for the simultaneous examination of the relationship between two correlated outcomes as they evolve while also identifying distinct subgroups within a heterogeneous population. Consistent with prior research, we performed the analysis in four sequential steps. First, we fitted single‐trajectory models for depressive severity and SEB to determine the optimal number of trajectories using a censored regular model (for a continuous outcome) and a logit model (for a binary outcome), respectively. Model building followed a two‐stage procedure: initially, we fitted several models sequentially to determine the appropriate number of classes. In the next step, we determined trajectory shapes considering constant, linear, quadratic, and cubic specifications. Models with increasing numbers of classes were compared using statistical (Bayesian Information Criterion [BIC] and Akaike’s Information Criterion [AIC]), substantive (entropy), and empirical (enough participants [>5%] occupied each class) criteria to determine the best‐fitting model. Second, we applied the dual‐trajectory model to examine the joint trajectories of depression and SEB. Third, we employed multivariable logistic regression to assess the associations between sociodemographic/clinical factors and dual trajectories. Finally, using a mixed‐effects linear model for repeated measures (MMRM), we evaluated the impact of varying SEB on changes in depression severity, adjusting for covariates identified in prior analyses.

Trajectory modeling was performed using the SAS TRAJ procedure [23], while all other data analyses were conducted using SAS software (Version 9.4; SAS Institute Inc., Cary, NC, USA). The statistical significance level was set to be 0.05 for this study. The completed STROBE checklist for cohort studies is provided in Supporting Information 2: File 1.

## 3. Results

In total, 1377 participants who initiated antidepressant treatment were included (Table 1). Among these participants, the mean age was 31.7 years (SD: 11.3), 71.3% were female, 32.0% had a bachelor’s degree or higher, and 62.9% were experiencing their first episode. For antidepressant treatment, 95.5% were monotherapy and 4.5% were a combination of antidepressants. From the sample, 1249 participants (91%) attended the week 4 assessment, 1047 (76%) attended the week 8 assessment, 819 (59%) attended the week 12 assessment, and 611 (44%) attended the week 24 assessment. The total score of QIDS‐SR16 at baseline was 16.5 (SD: 4.5) and 6.9 (SD: 6.1) at week 24.

**Table 1:** Patient characteristics.

Variables	Whole sample	No SEB	Late‐onset SEB	Early‐onset SEB	Persistent SEB
Sample size	1377	582 (41.5)	203 (14.7)	401 (29.1)	191 (13.9)
Age (*y*, mean [SD])	31.7 (11.3)	32.1 (11.4)	32.5 (11.6)	31.4 (11.2)	29.9 (11.1)
Gender (*n* [%])					
Male	395 (28.7)	181 (31.1)	49 (24.1)	120 (29.9)	45 (23.6)
Female	982 (71.3)	401 (68.9)	154 (75.9)	281 (70.1)	146 (76.4)
Education (*n* [%])					
Junior high or below	235 (17.1)	100 (17.2)	38 (18.7)	68 (17.0)	29 (15.2)
Senior high or junior college	702 (51.0)	184 (31.6)	79 (38.9)	118 (29.4)	59 (30.9)
University or above	440 (32.0)	298 (51.2)	86 (42.4)	215 (53.6)	103 (53.9)
Episode (*n* [%])					
First episode	866 (62.9)	382 (65.6)	124 (61.1)	252 (62.8)	108 (56.5)
Recurrence	511 (37.1)	200 (34.4)	79 (38.9)	149 (37.2)	83 (43.5)
Pharmacological treatment (*n* [%])					
Monotherapy	1315 (95.5)	562 (96.6)	195 (96.1)	386 (96.3)	172 (90.0)
SSRI	938 (68.1)	397 (68.2)	281 (70.1)	140 (69.0)	120 (62.8)
SNRI	238 (17.3)	99 (17.0)	37 (18.2)	66 (16.5)	36 (18.9)
Other antidepressant	139 (10.1)	66 (11.3)	18 (8.9)	39 (9.7)	16 (8.4)
Antidepressant combination	62 (4.5)	20 (3.4)	8 (3.9)	15 (3.7)	19 (10.0)
Total score of QIDS‐SR16 (mean [SD])	16.5 (4.5)	15.9 (4.5)	16.2 (4.3)	16.8 (4.3)	18.4 (4.6) ^∗^

*Note*: Monotherapy included SSRI, SNRI, and other antidepressants.

Abbreviations: QIDS‐SR16, 16‐item Quick inventory of depressive symptomatology‐self report; SD, standard deviation; SNRI, serotonin–norepinephrine reuptake inhibitor; SSRI, selective serotonin reuptake inhibitor.

^∗^ *p* < 0.05 (denotes significant differences in post‐hoc comparisons).

### 3.1. Trajectories in SEB Over Time

**Figure 1:**
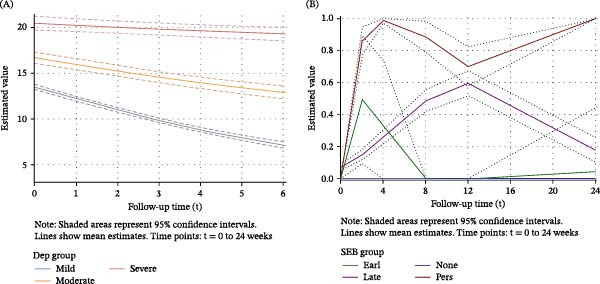
Estimated trajectories of depressive severity and side effect burden over 24 weeks. (A) Depressive severity trajectories over 24 weeks. (B) Side‐effect burden (SEB) trajectories over 24 weeks. The shaded areas represent 95% pointwise confidence intervals for the estimated trajectory groups.

Based on the criteria described above, a four‐class model was chosen for the SEB trajectories. The classes were interpreted as below: class 1: consistently no SEB (*N* = 582, 41.5%); class 2: early‐onset SEB (*N* = 401, 29.1%); class 3: late‐onset SEB (*N* = 203, 14.7%); class 4: persistent SEB (*N* = 191, 13.9%). Figure 1A depicts the trajectory model of SEB, and Supporting Information, 1: Table S2 summarizes the estimated values and 95% confidence intervals used to create this figure.

**Figure 2:**
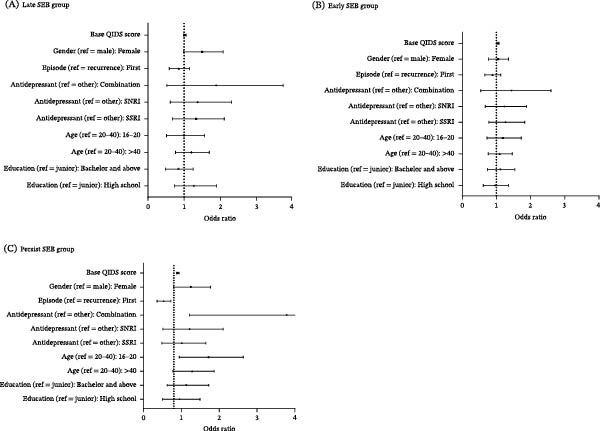
Forest plots of adjusted odds ratios comparing different side‐effect burden (SEB) trajectory groups. (A) Forest plot of the adjusted odds ratios for the association of indicators with the late‐SEB group and the no‐SEB group. (B) Forest plot of the adjusted odds ratios for the association of indicators with the early‐SEB group and the no‐SEB group. (C) Forest plot of the adjusted odds ratios for the association of indicators with the persistent SEB group and the no‐SEB group.

Similar distributions of age, gender, education level, episode type, and pharmacological treatment patterns were observed across all four SEB classes (all *p* values > 0.05). In comparison, the persistent class demonstrated significantly higher baseline depression severity as measured by QIDS‐SR16 scores of 18.4 (SD: 4.6). Figure 2 and Supporting Information 1: Table S3 represent multivariate logistic regression results comparing three SEB trajectory classes (late, early, and persistent) compared to the no‐SEB class as a reference. Several significant associations were identified, including baseline QIDS‐SR16 scores, which showed the strongest association observed for persistent SEB vs. no SEB (OR = 1.13, 95% CI: 1.08–1.17). The first episode of depression was significantly associated with lower odds of persistent SEB (OR = 0.63, 95% CI: 0.44–0.90). Pharmacologically, combination antidepressant therapy increased the odds of persistent SEB by 3.7 times (95% CI: 1.52–9.02).

### 3.2. Trajectories in Depressive Severity Over Time

Figure 1B plots the trajectory model of depressive severity (see Supporting Information 1: Table S2 for details), primarily characterized by baseline severity and degree of prognosis, demonstrating three distinct change trajectories among patients across 24 weeks: mild‐responsive group (*N* = 917, 66.6%), moderate‐progressive group (*N* = 258, 18.7%), and chronic‐severe group (*N* = 202, 14.7%).

### 3.3. Dual Trajectories in SEB and Depression Over Time

Table 2 presents the dual trajectories and reveals a significant co‐occurrence pattern between SEB and depressive trajectories (*p* < 0.001).

**Table 2:** Dual trajectories of side‐effect burden and depressive severity.

2A. The probability of membership in each depression severity group conditional on the side‐effect group				
Side‐effect group	Depression severity group			Conditional probability
No side‐effect burden	Mild and remit			84.8
	Moderate and remit			15.2
	Severe			0.0
Late side‐effect burden	Mild and remit			60.9
	Moderate and remit			20.8
	Severe			18.3
Early side‐effect burden	Mild and remit			73.1
	Moderate and remit			19.1
	Severe			7.7
Persistent side‐effect burden	Mild and remit			2.4
	Moderate and remit			43.6
	Severe			54.0
2B. The probability of membership in each side‐effect group conditional on the depression severity group				
Depression severity group	Side‐effect group			Conditional probability
Mild and remit	No side‐effect burden			25.5
	Late side‐effect burden			25.4
	Early side‐effect burden			48.5
	Persistent side‐effect burden			0.5
Moderate and remit	No side‐effect burden			12.9
	Late side‐effect burden			24.5
	Early side‐effect burden			35.9
	Persistent side‐effect burden			26.7
Severe	No side‐effect burden			0.0
	Late side‐effect burden			31.1
	Early side‐effect burden			21.0
	Persistent side‐effect burden			47.8
2C. Dual probability of depression and side‐effect burden				
Side‐effect group	Mild	Moderate		Severe
No side‐effect burden	16.0	2.9		0.0
Late side‐effect burden	15.9	5.4		4.8
Early side‐effect burden	30.4	8.0		3.2
Persistent side‐effect burden	0.3	5.9		7.3

*Note:* Mild, mild ‐responsive; Moderate, moderate ‐progressive; Severe, chronic‐severe.

First, conditional probabilities revealed symptom severity‐dependent SEB patterns (Table 2A,B). Patients who did not experience SEB, experienced early‐onset SEB, or tolerated late‐onset SEB were predominantly distributed among patients with mild severity, with proportions of 84.8%, 73.1%, and 60.9%, respectively. Patients who experienced persistent SEB primarily presented with moderate or severe depressive symptoms (Table 2A).

Second, regarding severity trajectory, 25.5% of patients with mild severity did not experience a SEB. Nearly 48.5% and 25.4% of patients experienced an adverse effect burden at the beginning and late stages of treatment, respectively, but ultimately tolerated it. As the severity of the disease increased, the proportion of patients reporting no adverse effect burden decreased progressively, while the proportion of patients with persistent adverse effect burden increased. Over 24 weeks, 47.8% of patients with chronic –severe disease had a persistent SEB (Table 2B).

Finally, the joint membership probabilities revealed that 30.4% displayed mild depressive severity and early‐onset SEB; 16.0% and 15.9% of participants displayed mild depressive severity with either no SEB or late‐onset SEB; and other combinations accounted for a small proportion of the sample. Notably, 7.3% exhibited concurrent chronic‐severe depressive severity and persistent SEB, while no participants with severe depression occurred without concurrent SEB.

### 3.4. Effect of SEB on Treatment Outcome

MMRM models revealed significant differences in QIDS‐SR16 scores (all *p*  < 0.001 except late‐onset SEB vs. early‐onset SEB). The persistent group showed significantly higher scores (worse outcomes) than all others (vs. no SEB: 4.15, 95% CI: 3.30, 5.00; vs. early‐onset SEB: −2.90, 95% CI: −3.79, −2.01; vs. late‐onset SEB: −3.00, 95% CI: −3.98, −2.01). The no‐SEB group demonstrated the best outcomes, with outcomes significantly lower than those of all groups (all *p* < 0.001). No difference existed between late‐onset SEB and early‐onset‐SEB groups (*p* = 0.991) (Table 3).

**Table 3:** Comparisons of changes in QIDS‐SR16 at week 24, change from baseline between SEB groups.

Comparisons	QIDS‐SR16 mean changes (adjusted 95% CI)	Adjusted *p*
Late vs. early	−0.09 (−0.92, 0.73)	0.9914
Late vs. persist	−3.00 (−3.98, −2.01)	<0.001
Late vs. no	1.15 (0.36,1.94)	0.0010
Early vs. persist	−2.90 (−3.79, −2.01)	<0.001
Early vs. no	1.24 (0.58, 1.91)	<0.001
Persist vs. no	4.15 (3.30, 5.00)	<0.001

*Note:* Comparisons are expressed as the first SEB group minus the second SEB group. Positive values indicate less reduction in QIDS‐SR16 scores in the first group. Early, Early‐onset SEB; Late, Late‐onset SEB; No, No SEB; Persist, Persistent SEB.

Abbreviations: CI, confidence interval; QIDS‐SR16, 16‐item quick inventory of depressive symptomatology‐self report; SEB, side‐effect burden.

### 3.5. Associated Factors With Co‐Trajectories of Depressive Severity and SEB

Our primary focus was to compare the two groups diagonally. In the logistic regression analysis, we explored associated factors with co‐occurring severe depressive severity and persistent SEB as the outcome (based on the most likely posterior membership), with patients experiencing mild depressive severity and no SEB as the control group. The analysis revealed that female patients (OR = 1.95, 95% CI: 1.11–3.42) and younger patients (OR = 5.02, 95% CI: 2.63–9.55) were more likely to experience greater severity and a greater SEB. Higher education level was positively associated with the probability of experiencing severe symptoms and SEB (high school: OR = 2.5, 95% CI: 1.12–5.36; bachelor’s degree or higher: OR = 2.68, 95% CI: 1.24–5.82).

Regarding clinical characteristics and pharmacological treatment, the first episode of depression was significantly associated with lower odds of severity and SEB (OR = 0.54, 95% CI: 0.33–0.86). The risk of experiencing chronic‐severe symptoms and SEB was higher for those who use antidepressant combinations compared with other types of antidepressants (OR = 8.27, 95% CI: 2.69–25.45), while neither SSRIs (OR = 1.06, 95% CI: 0.46–2.44) nor SNRIs (OR = 1.37, 95% CI: 0.44–2.96) were associated with severity and SEB.

## 4. Discussion

The current study investigated 6‐month longitudinal trajectories of SEB and its interaction with depressive severity during antidepressant treatment among a large sample of patients with MDD. Contrary to previous studies that focused on treatment adherence, used limited data collected at short intervals, or ignored the dynamic association with depressive severity, the present study included six data points over 24 weeks and their temporal relationships. Overall, this study identified four distinct SEB trajectories: no SEB, early‐onset SEB, late‐onset SEB, and persistent SEB. Patients who developed tolerance to antidepressant side effects achieved comparable improvement in disease severity to those without side effects, regardless of whether the burden emerged early or late during treatment. Conversely, the persistent SEB, reflecting failed tolerance acquisition, was significantly associated with adverse clinical outcomes.

Contrary to the common clinical assumption that antidepressant side‐effect complaints improve with continued treatment, our findings reveal that 13.9% of patients experienced persistent SEB throughout the 6‐month follow ‐up, with this trajectory group exhibiting significantly poorer depression outcomes compared to those with transient or no SEB. These results align with emerging evidence from the iSPOT ‐D, which documented sustained side effects during prolonged antidepressant use [7]. Significantly, our trajectory‐based approach extends prior cross‐sectional or short‐term studies by delineating temporal patterns, thereby capturing heterogeneity in patients’ tolerability profiles that static measures fail to detect. Notably, the identification of a late‐onst SEB trajectory (14.7%) further complicates the endurance narrative, suggesting that a delayed onset of burdensome side effects may represent a previously unrecognized vulnerability factor for nonadherence or treatment discontinuation [24]. Although patients with documented poor adherence were excluded from this analysis, real‐world clinical practice often lacks systematic monitoring of side effects. Implementation of dynamic assessment tools (e. g., digital symptom trackers) could facilitate earlier detection and intervention for emerging SEB. Our dual‐trajectory analyses revealed a severity gradient wherein persistent SEB disproportionately co‐occurred with severe depressive trajectories, while mild/moderate depression primarily coincided with no or transient SEB.

The absence of severe depression cases without concurrent SEB further suggests a close co‐occurrence between SEB and therapeutic response, which is consistent with clinical frameworks in which SEB may serve as both a contributor to and a consequence of poor treatment efficacy [25]. The lack of significant differences in outcomes between early‐onset and late‐onset SEB groups suggests that overall burden duration may be more critical than the timing of SEB onset. This finding argues for continuous monitoring throughout the treatment.

Our findings highlight a distinct clinical phenotype characterized by co‐occurring chronic‐severe depressive symptoms and persistent SEB, particularly prevalent among female, younger, and highly educated patients, as well as those receiving antidepressant combination therapy. The observed gender difference is consistent with pharmacological evidence indicating a higher overall risk of antidepressant adverse drug reactions in women [26]. This difference may reflect drug metabolism and estrogen‐mediated modulation of serotonin signaling (5‐HT1A) [27], which may influence antidepressant efficacy and clinical response across sexes [28].

Regarding behavioral and psychosocial factors, both younger age and higher education level appear to influence treatment outcomes through adherence and perceptions. Specifically, younger age is often associated with poorer treatment [29] and also presents distinct symptom profiles and comorbidity patterns, which can shape symptom attribution and side‐effect ratings [30]. Similarly, while highly educated patients may also exhibit unique adherence patterns, their increased SEB is often attributed to heightened self‐monitoring [31, 32] and work‐related stress. Moreover, the ongoing maturation of the prefrontal cortex in younger patients may further sensitize them to serotonergic modulation [33].

Conversely, first‐episode patients had lower odds of SEB, possibly due to greater neuroplasticity reserves [34] than those with recurrent illness. Notably, antidepressant polypharmacy conferred the highest risk, likely due to pharmacokinetic interactions (e. g., CYP450 inhibition) and clinically significant interactions (e. g., serotonin syndrome and hypertensive emergencies), underscoring the need for cautious combination strategies [35, 36]. These findings emphasize the interplay between demographic, pharmacogenetic, and neurobiological factors in shaping antidepressant outcomes. Further studies should integrate multiomics profiling to unravel biomarkers for high‐risk subgroups, enabling precision prescribing strategies.

### 4.1. Clinical Implications

The most direct clinical implication of our findings is the potential to support early risk stratification. For example, a clinician encountering a patient with severe baseline depression, a history of prior episodes, or receiving combination therapy can now more accurately identify those at a high risk of following a “Persistent SEB” or “Severe” depression trajectory, thereby allowing for more proactive and targeted monitoring. This knowledge should trigger proactive management.

Proactive management could include (1) more frequent follow‐up visits in the first month; (2) explicitly setting expectations about potential side effects and creating a collaborative plan to manage them; (3) considering an initial antidepressant choice with a potentially more benign side‐effect profile for that specific patient; and (4) Quicker consideration of dose adjustment or switching if side effects are burdensome.

Finally, while our study constructed a reliable clinical model, future research can build upon this by integrating biological measures. Identifying the pharmacogenomic or therapeutic drug monitoring associated with the high‐risk trajectories we have defined could further refine these predictive models and eventually lead to biomarker‐guided interventions.

### 4.2. Limitations and Strengths

However, the findings of the present study must be interpreted in the context of several limitations. First, while the use of a single‐item measure for SEB may provide less detail regarding specific domains, it was a pragmatic choice that prioritized high participant response rates and captured the functional impact of side effects on patients. Second, the exclusion of patients with low adherence may limit the generalizability of the findings. Since those who discontinue treatment due to intolerable side effects were not included, our estimates of persistent SEB should be viewed as conservative, likely underestimating the true burden encountered in routine clinical practice. Third, the study did not account for potential factors, such as medication dosage and comorbidities, that might influence side‐effect trajectories and treatment outcomes. Moreover, the study did not systematically assess specific types of side effects (e. g., gastrointestinal, neurological, or sexual dysfunction), which may have distinct temporal patterns and clinical implications. Future research incorporating detailed side‐effect categorization could further refine our understanding of their differential impact on treatment outcomes.

Despite the above limitations, this study has worth‐mentioning strengths. It employs a robust longitudinal design with six data points over 6 months, allowing for the examination of dynamic side‐effect trajectories and their interactions with depressive severity. The use of a dual‐trajectory model provides a nuanced understanding of heterogeneous patient subgroups, which static measures cannot achieve. Additionally, the large multicenter cohort enhances the generalizability of findings across diverse clinical settings in China. The study also highlights the clinical significance of persistent SEB, offering valuable insights for personalized treatment strategies. These findings underscore the need for proactive monitoring and timely interventions to improve antidepressant tolerability and outcomes.

## 5. Conclusion

This study advances understanding of antidepressant SEB as a dynamic, heterogeneous phenomenon with direct clinical consequences. Persistent SEB, observed in 14% of patients, is a critical predictor of poor outcomes, highlighting the need to shift from a uniform expectation of side‐effect tolerance to personalized management. By integrating SEB monitoring into routine care, clinicians can optimize adherence and improve remission rates in MDD.

## Author Contributions

**Xuequan Zhu**: conceptualization, methodology, validation, formal analysis, visualization, writing – original draft, writing – review and editing. **Kaiyan Gan**: conceptualization, methodology, data curation, writing – review and editing. **Xu Chen**: methodology, investigation, data curation. **Lei Feng**: methodology, investigation, formal analysis. **Yuan Feng**: investigation, data curation. **Ling Zhang**: investigation, formal analysis. **Gang Wang**: conceptualization, methodology, resources, supervision, project administration, funding acquisition, writing – review and editing. All authors have actively contributed to the study’s design, execution, and analysis processes.

## Funding

This work was supported by Capital’s Funds for Health Improvement and Research (Grant 2026‐2‐2128) and the Beijing Municipal Science & Technology Commission (Grant Z221100007422010).

## Conflicts of Interest

The authors declare no conflicts of interest.

## Supporting Information

Additional supporting information can be found online in the Supporting Information section.

## Supporting information


**Supporting Information 1** Table S1: Shows the item descriptions of side‐effect burden at baseline and each point of the follow‐up period. Table S2: Shows the estimated values and 95% confidence intervals of SEB and depression trajectory groups. Table S3: Shows the multivariate logistic regression results comparing three SEB trajectory classes with the no‐SEB class as reference.

**Supporting Information 2** File 1: Completed STROBE checklist for cohort studies.

## Data Availability

The data that support the findings of this study are available from the corresponding author upon reasonable request.
