# Supplementary material for: Identifying Clinical Predictors of Unfavorable Treatment Trajectories in Major Depressive Disorder: A National Multicentered Prospective Cohort Longitudinal, Naturalistic Study
Source: Depress Anxiety. 2026 Jul 17;2026:9897990. doi: 10.1155/da/9897990 (PMC13377403; doi:10.1155/da/9897990)
Supplement: Supplementary file 1 — Supporting Information 1 Table S1: Shows the item descriptions of side‐effect burden at baseline and each point of the follow‐up period. Table S2: Shows the estimated values and 95% confidence intervals of SEB and depression trajectory groups. Table S3: Shows the multivariate logistic regression results comparing three SEB trajectory classes with the no‐SEB class as reference. [file DA-2026-9897990-s002.docx]

**Table S1. Side effect burden rated by participants**

| Score | Description：Choose the response that best describes the degree to which antidepressant medication side effects that you have had  over the last week have interfered with your day-to-day functions. |
| --- | --- |
| 1 | No impairment |
| 2 | Minimal impairment |
| 3 | Mild impairment |
| 4 | Moderate impairment |
| 5 | Marked impairment |
| 6 | Severe impairment |
| 7 | Unable to function |

**Table S2. Estimated mean values (95% CI) across study time points for SEB and depression trajectory groups.**

| **Time**  **(week)** | **Depressive Severity** | | **Side Effect Burden** | |
| --- | --- | --- | --- | --- |
|  | **Group** | **Estimate(95%CI)** | **Group** | **Estimate(95%CI)** |
| 0 | Mild and remit | 13.48(13.22-13.74) | Early SEB | 0.00(0.00-0.00) |
| 2 | Mild and remit | 10.96(10.75-11.18) | Early SEB | 0.49(0.00-1.00) |
| 4 | Mild and remit | 8.78(8.54-9.01) | Early SEB | 0.33(0.00-1.00) |
| 8 | Mild and remit | 5.47(5.11-5.83) | Early SEB | 0.00(0.00-0.00) |
| 12 | Mild and remit | 3.60(3.21-3.98) | Early SEB | 0.00(0.00-0.00) |
| 24 | Mild and remit | 4.27(3.70-4.84) | Early SEB | 0.04(0.00-1.00) |
| 0 | Moderate and remit | 16.72(16.10-17.33) | Late SEB | 0.07(0.05-0.09) |
| 2 | Moderate and remit | 15.25(14.70-15.79) | Late SEB | 0.15(0.12-0.18) |
| 4 | Moderate and remit | 13.94(13.33-14.55) | Late SEB | 0.26(0.22-0.30) |
| 8 | Moderate and remit | 11.83(11.00-12.66) | Late SEB | 0.49(0.42-0.56) |
| 12 | Moderate and remit | 10.40(9.38-11.43) | Late SEB | 0.60(0.52-0.67) |
| 24 | Moderate and remit | 10.15(8.73-11.56) | Late SEB | 0.18(0.10-0.25) |
| 0 | Severe | 20.47(19.70-21.25) | None SEB | 0.00(0.00-0.00) |
| 2 | Severe | 20.03(19.38-20.68) | None SEB | 0.00(0.00-0.00) |
| 4 | Severe | 19.63(18.97-20.29) | None SEB | 0.00(0.00-0.00) |
| 8 | Severe | 18.96(18.16-19.75) | None SEB | 0.00(0.00-0.00) |
| 12 | Severe | 18.46(17.59-19.33) | None SEB | 0.00(0.00-0.00) |
| 24 | Severe | 18.07(16.96-19.19) | None SEB | 0.00(0.00-0.00) |
| 0 | / | / | Persistent SEB | 0.00(0.00-0.00) |
| 2 | / | / | Persistent SEB | 0.86(0.77-0.95) |
| 4 | / | / | Persistent SEB | 0.99(0.97-1.00) |
| 8 | / | / | Persistent SEB | 0.89(0.79-0.98) |
| 12 | / | / | Persistent SEB | 0.70(0.57-0.82) |
| 24 | / | / | Persistent SEB | 1.00(1.00-1.00) |

**Table S3. Multivariate Logistic regression model**

|  | **Late vs None** | | | | | | | **Early vs None** | | | | | | | **Persistent vs None** | | | | | | |
| --- | --- | --- | --- | --- | --- | --- | --- | --- | --- | --- | --- | --- | --- | --- | --- | --- | --- | --- | --- | --- | --- |
|  | β | SE | χ^2^ | P | OR | 95% CI | | β | SE | χ^2^ | P | OR | 95% CI | | β | SE | χ^2^ | P | OR | 95% CI | |
| **Intercept** | -1.357 | 0.350 | 15.064 | 0.000 |  |  |  | -1.109 | 0.285 | 15.139 | <.0001 |  |  |  | -2.843 | 0.392 | 52.698 | <.0001 |  |  |  |
| **Baseline QIDS-SR16** | 0.017 | 0.019 | 0.819 | 0.366 | 1.02 | 0.98 | 1.06 | 0.045 | 0.015 | 8.924 | 0.003 | 1.05 | 1.02 | 1.08 | 0.119 | 0.021 | 33.710 | <.0001 | 1.13 | 1.08 | 1.17 |
| **Female(ref=male)** | 0.179 | 0.095 | 3.584 | 0.058 | 1.43 | 0.99 | 2.08 | 0.012 | 0.072 | 0.026 | 0.871 | 1.02 | 0.77 | 1.36 | 0.196 | 0.102 | 3.690 | 0.055 | 1.48 | 0.99 | 2.21 |
| **First episode** | -0.099 | 0.085 | 1.345 | 0.246 | 0.82 | 0.59 | 1.15 | -0.074 | 0.069 | 1.163 | 0.281 | 0.86 | 0.66 | 1.13 | -0.229 | 0.090 | 6.400 | 0.011 | 0.63 | 0.44 | 0.90 |
| **Antidepressant(ref=other)** | | | | |  |  |  |  |  |  |  |  |  |  |  |  |  |  |  |  |  |
| **Combination** | 0.160 | 0.329 | 0.236 | 0.627 | 1.40 | 0.52 | 3.73 | 0.053 | 0.268 | 0.039 | 0.844 | 1.19 | 0.54 | 2.60 | 0.889 | 0.277 | 10.305 | 0.001 | 3.70 | 1.52 | 9.02 |
| **SNRI** | 0.004 | 0.197 | 0.001 | 0.983 | 1.20 | 0.62 | 2.31 | 0.004 | 0.161 | 0.001 | 0.980 | 1.13 | 0.68 | 1.89 | -0.160 | 0.195 | 0.675 | 0.411 | 1.30 | 0.64 | 2.62 |
| **SSRI** | 0.009 | 0.155 | 0.004 | 0.951 | 1.20 | 0.68 | 2.11 | 0.061 | 0.124 | 0.240 | 0.624 | 1.20 | 0.78 | 1.83 | -0.310 | 0.149 | 4.319 | 0.038 | 1.12 | 0.60 | 2.06 |
| **Age categories(ref=20-40)** | | | | |  |  |  |  |  |  |  |  |  |  |  |  |  |  |  |  |  |
| **16-20** | -0.115 | 0.189 | 0.368 | 0.544 | 0.90 | 0.51 | 1.57 | 0.057 | 0.147 | 0.151 | 0.698 | 1.12 | 0.73 | 1.73 | 0.316 | 0.173 | 3.338 | 0.068 | 1.97 | 1.18 | 3.29 |
| **≥40** | 0.121 | 0.150 | 0.653 | 0.419 | 1.14 | 0.76 | 1.69 | 0.000 | 0.121 | 0.000 | 0.997 | 1.06 | 0.76 | 1.47 | 0.046 | 0.154 | 0.088 | 0.767 | 1.50 | 0.97 | 2.33 |
| **Education level (ref=junior school)** | | | | |  |  |  |  |  |  |  |  |  |  |  |  |  |  |  |  |  |
| **Bachelor and above** | -0.218 | 0.119 | 3.359 | 0.067 | 0.78 | 0.49 | 1.25 | 0.073 | 0.094 | 0.614 | 0.433 | 1.07 | 0.74 | 1.54 | 0.146 | 0.126 | 1.339 | 0.247 | 1.29 | 0.78 | 2.15 |
| **High school** | 0.192 | 0.120 | 2.558 | 0.110 | 1.18 | 0.74 | 1.89 | -0.082 | 0.102 | 0.659 | 0.417 | 0.91 | 0.62 | 1.35 | -0.035 | 0.138 | 0.063 | 0.802 | 1.08 | 0.63 | 1.86 |
