# Supplementary material for: Identifying Clinical Predictors of Unfavorable Treatment Trajectories in Major Depressive Disorder: A National Multicentered Prospective Cohort Longitudinal, Naturalistic Study
Source: Depress Anxiety. 2026 Jul 17;2026:9897990. doi: 10.1155/da/9897990 (PMC13377403; doi:10.1155/da/9897990)
Supplement: Supplementary file 2 — Supporting Information 2 File 1: Completed STROBE checklist for cohort studies. [file DA-2026-9897990-s001.doc]

STROBE Statement—Checklist of items that should be included in reports of ***cohort studies***

|  | Item No | Recommendation | Page  No. | | Relevant text from manuscript | |
| --- | --- | --- | --- | --- | --- | --- |
| **Title and abstract** | 1 | (*a*) Indicate the study’s design with a commonly used term in the title or the abstract | Pages 1 | | Title: “a national multi-centered prospective cohort longitudinal, naturalistic study” | |
|  |  | (*b*) Provide in the abstract an informative and balanced summary of what was done and what was found | Pages 2–3 | | The Abstract summarizes Background, Methods, Results, and Conclusion, including sample, assessment time points, measures, trajectory modeling, key ORs, and clinical implications. | |
| Introduction | | |  | |  | |
| Background/rationale | 2 | Explain the scientific background and rationale for the investigation being reported | Pages 4–5 | | Introduction describes antidepressant efficacy and tolerability, real-world side effect burden, lack of longitudinal data on temporal evolution, and the neglected interaction between evolving side effects and treatment outcomes. | |
| Objectives | 3 | State specific objectives, including any prespecified hypotheses | Page 5 | | Introduction: “We aimed to first characterize the joint trajectories of depressive severity and side effect burden(SEB) as they evolve together during the initial months of antidepressant treatment.” | |
| Methods | | |  | |  | |
| Study design | 4 | Present key elements of study design early in the paper | Page 5 | | 2.1 Participants and settings: “The current study pooled data from three studies conducted between 2015 and 2022, all of which enrolled patients with MDD initiating antidepressant treatment for their current episode and implemented similar eligibility criteria and follow-up protocols:…” | |
| Setting | 5 | Describe the setting, locations, and relevant dates, including periods of recruitment, exposure, follow-up, and data collection | Pages 5–6 | | Described for three studies: April 2015 to December 2017 at 11 psychiatric hospitals or units in general hospitals in 9 cities; September 2016 to December 2020 at eight psychiatric hospitals or units in general hospitals in 8 cities; August 2020 to December 2022 at 17 psychiatric hospitals and 10 general units in general hospitals in 19 cities. Follow-up at baseline and weeks 2, 4, 8, 12, and 24. | |
| Participants | 6 | (*a*) Give the eligibility criteria, and the sources and methods of selection of participants. Describe methods of follow-up | Page 6 | | Eligibility criteria included outpatients aged ≥18 years, depressive episodes, recurrent depressive disorder based on DSM-5 or ICD-10, initiating treatment for current episode, and moderate or severer symptoms. Patients with low adherence were excluded; patients with at least week 2 follow-up were included. | |
|  |  | (*b*) For matched studies, give matching criteria and number of exposed and unexposed | N/A | | Not applicable: this was not a matched cohort study. | |
| Variables | 7 | Clearly define all outcomes, exposures, predictors, potential confounders, and effect modifiers. Give diagnostic criteria, if applicable | Pages 6–8 | | QIDS-SR16 for depressive severity; FIBSER burden item for SEB; pharmacological treatment categories; sociodemographic/clinical factors and dual trajectories in logistic regression. | |
| Data sources/ measurement | 8* | For each variable of interest, give sources of data and details of methods of assessment (measurement). Describe comparability of assessment methods if there is more than one group | Pages 6–7 | | QIDS-SR16 completed at baseline and each follow-up; SEB indexed by the third FIBSER item and measured at baseline and each follow-up; pharmacological treatment recorded at baseline and each follow-up visit. | |
| Bias | 9 | Describe any efforts to address potential sources of bias | Pages 7, 14–15 | | Dichotomization of SEB “was used to ensure comparability across the pooled naturalistic cohorts”; limitations discuss single-item SEB measurement, exclusion of low-adherence patients, and unmeasured factors such as medication dosage and comorbidities. | |
| Study size | 10 | Explain how the study size was arrived at | Pages 6, 8 | | “In the current study, we included patients with at least a week 2 follow-up assessment”; Results: “In total, 1377 participants who initiated antidepressant treatment were included”. | |
| Quantitative variables | 11 | Explain how quantitative variables were handled in the analyses. If applicable, describe which groupings were chosen and why | Pages 6–8, 21 | | QIDS-SR16 total score ranged from 0 to 27; SEB dichotomized as mild impairment or severer = 1, and no or minimal impairment = 0; pharmacological treatment categorized as SSRI, SNRI, Other antidepressants, and antidepressant combinations; trajectory class selection used BIC, AIC, entropy, and minimum class size (>5%). | |
| Statistical methods | 12 | (*a*) Describe all statistical methods, including those used to control for confounding | Pages 7–8 | | Statistical methods include descriptive statistics, ANOVA/Newman–Keuls tests, CMH tests, dual-trajectory modeling, multivariable logistic regression, and MMRM; trajectory modeling used SAS TRAJ and other analyses used SAS 9.4. | |
|  |  | (*b*) Describe any methods used to examine subgroups and interactions | Pages 8, 11 | | Methods report multivariable logistic regression assessing associations between sociodemographic/clinical factors and dual trajectories; Results compare chronic-severe depression with persistent SEB versus mild depression with no SEB. | |
|  |  | (*c*) Explain how missing data were addressed | Pages 8–9 | | Results reports eligibility and follow-up counts. | |
|  |  | (*d*) If applicable, explain how loss to follow-up was addressed | Pages 6, 8–9 | | Scheduled follow-up assessments were at baseline and weeks 2, 4, 8, 12, and 24; Results report attendance at weeks 4, 8, 12, and 24. | |
|  |  | (*e*) Describe any sensitivity analyses | — | | — | |
| Results | | |  | |  | |
| Participants | 13* | (a) Report numbers of individuals at each stage of study—eg numbers potentially eligible, examined for eligibility, confirmed eligible, included in the study, completing follow-up, and analysed | Pages 6, 8–9, 21 | | Participants included those with at least week 2 follow-up; Results report N = 1377 included and 1249 participants attended the week 4, 1047 attended the week 8, 819 attended the week 12, and 611 attended the week 24t.; Table 1 reports whole sample and SEB trajectory group sample sizes. | |
|  |  | (b) Give reasons for non-participation at each stage | — | | — | |
|  |  | (c) Consider use of a flow diagram | — | | — | |
| Descriptive data | 14* | (a) Give characteristics of study participants (eg demographic, clinical, social) and information on exposures and potential confounders | Pages 8–9, 21 | | Results and Table 1 provide participant characteristics: age, gender, education, episode type, pharmacological treatment, and baseline QIDS-SR16 by whole sample and SEB trajectory groups. | |
|  |  | (b) Indicate number of participants with missing data for each variable of interest | Pages 8–9 | | Results reports eligibility and follow-up counts. | |
|  |  | (c) Summarise follow-up time (eg, average and total amount) | Pages 2, 6, 8–9, 15 | | Follow-up covered baseline and weeks 2, 4, 8, 12, and 24; Results report week-specific attendance and QIDS-SR16 values at baseline and week 24; Discussion describes six data points over six months. | |
| Outcome data | 15* | Report numbers of outcome events or summary measures over time | Pages 9–11, 23–25 | | Results report SEB trajectory membership, depressive severity trajectory membership, dual trajectory probabilities, MMRM comparisons, and associated factors; Tables 2 and 3 summarize dual probabilities and adjusted mean changes. | |
| Main results | 16 | (*a*) Give unadjusted estimates and, if applicable, confounder-adjusted estimates and their precision (eg, 95% confidence interval). Make clear which confounders were adjusted for and why they were included | Pages 8–11, 25 | | Methods state MMRM adjusted for covariates identified in prior analyses; Results report adjusted ORs with 95%CI for SEB and co-trajectory analyses and adjusted mean changes with 95%CI in Table 3. | |
|  |  | (*b*) Report category boundaries when continuous variables were categorized | Pages 6–9, 21, 23 | | Category boundaries are reported for dichotomized SEB (mild impairment or severer vs no impairment), pharmacological treatment categories, and trajectory classes/proportions. | |
|  |  | (*c*) If relevant, consider translating estimates of relative risk into absolute risk for a meaningful time period | N/A | | Not applicabl. | |
| Other analyses | 17 | Report other analyses done—eg analyses of subgroups and interactions, and sensitivity analyses | Pages 9–11, 23–25 | | Other analyses include SEB trajectories, depressive severity trajectories, dual trajectory probabilities, MMRM comparisons, and logistic regression for co-occurring chronic-severe depression and persistent SEB. | |
| Discussion | | |  | |  | |
| Key results | 18 | Summarise key results with reference to study objectives | Pages 11–12, 15 | | Discussion: The study identified four SEB trajectories and that persistent SEB was significantly associated with adverse clinical outcomes. Conclusion: persistent SEB is a critical predictor of poor outcomes. | |
| Limitations | 19 | Discuss limitations of the study, taking into account sources of potential bias or imprecision. Discuss both direction and magnitude of any potential bias | Pages 14–15 | | Limitations: The single-item SEB measure, exclusion of low-adherence patients, lack of medication dosage and comorbidities data, and absence of systematic assessment of specific side effects types. The bias is discussed as conservative underestimation of persistent SEB. | |
| Interpretation | 20 | Give a cautious overall interpretation of results considering objectives, limitations, multiplicity of analyses, results from similar studies, and other relevant evidence | Pages 12–15 | | Interpretation considers objectives and relevant evidence: persistent SEB challenges the clinical expectation that side effects diminish with treatment; clinical implications and limitations are discussed. | |
| Generalisability | 21 | Discuss the generalisability (external validity) of the study results | Page 15 | | Exclusion of low-adherence patients may limit generalizability, while the large multicenter cohort enhances generalizability across diverse clinical settings in China. | |
| Other information | | | |  | |  |
| Funding | 22 | Give the source of funding and the role of the funders for the present study and, if applicable, for the original study on which the present article is based | Page 16 | | Funding: “This work was supported by Beijing Municipal Science & Technology Commission (Z221100007422010).” | |

*Give information separately for exposed and unexposed groups.

**Note:** An Explanation and Elaboration article discusses each checklist item and gives methodological background and published examples of transparent reporting. The STROBE checklist is best used in conjunction with this article (freely available on the Web sites of PLoS Medicine at http://www.plosmedicine.org/, Annals of Internal Medicine at http://www.annals.org/, and Epidemiology at http://www.epidem.com/). Information on the STROBE Initiative is available at http://www.strobe-statement.org.
